# Copper homeostasis in macrophages: regulatory mechanisms and immunological implications

**DOI:** 10.3389/fchem.2026.1924097

**Published:** 2026-09-07

**Authors:** Wanting Yang, Junfeng Wang, Zhenbin Dong, Fangming Zhang, Guofeng Li, Xing Wang, Wensheng Xie

**Affiliations:** The State Key Laboratory of Organic-Inorganic Composites, The Beijing Laboratory of Biomedical Materials, College of Life Science and Technology, Beijing University of Chemical Technology, Beijing, China

**Keywords:** copper homeostasis, immunological regulation, inflammation, macrophage polarization, molecular mechanisms

## Abstract

Copper is an essential micronutrient whose redox activity underpins a dual role in immunity: it serves as both an antimicrobial effector and a regulator of inflammatory signaling. Macrophages, as central orchestrators of innate and adaptive immunity, maintain sophisticated copper homeostasis mechanisms that dynamically adapt to distinct activation states and environmental cues. This review synthesizes current knowledge around three interconnected themes: (1) the molecular machinery governing copper transport and regulation—including CTR1, ATP7A, ATP7B, and copper chaperones; (2) the functional interplay between copper metabolism, macrophage polarization, and immunometabolism; and (3) the pathophysiological consequences of copper dysregulation in infection, chronic inflammation, and cancer. Emerging evidence reveals that copper exerts dose-dependent effects on macrophage polarization: low-to-moderate copper promotes an anti-inflammatory M2 phenotype via STAT6 and PI3K/Akt pathways, whereas high copper concentrations trigger oxidative stress and NF-κB activation, driving pro-inflammatory M1 polarization. Furthermore, recent findings highlight crosstalk among copper metabolism, cuproptosis, and tumor-associated macrophage function, opening new avenues for copper-based immunotherapy. This review identifies critical knowledge gaps—including tissue-specific copper regulation, single-cell dynamics of copper trafficking, and the therapeutic potential of copper-targeted interventions. While acknowledging the bidirectional causality between copper metabolism and macrophage activation, we argue that copper homeostasis functions as a rheostat of immune competence with substantial translational promise.

## 1 Introduction

Copper is an essential redox-active transition metal that participates in critical biological processes, including mitochondrial respiration, antioxidant defense, and neurotransmitter biosynthesis (Chen et al., 2022). Its ability to shuttle between Cu(I) and Cu(II) states enables Fenton-type reactions that generate reactive oxygen species (ROS), rendering copper both an indispensable cofactor for enzymes such as superoxide dismutase 1 (SOD1) and a potential toxin when present in excess (Xie et al., 2023). The immune system exploits this duality: copper is concentrated in phagosomes to kill ingested pathogens while also regulating inflammatory signaling pathways in the cytoplasm and nucleus (Craciun et al., 2025). This dual role creates a delicate balance—insufficient copper impairs antimicrobial defense, whereas excessive copper drives pathological inflammation and tissue damage. Unchaperoned free copper induces cytotoxicity via excessive ROS production, leading to oxidative damage to lipids, proteins, and nucleic acids (Jiang et al., 2025). Copper deficiency suppresses macrophage activity and inflammatory cytokine production, while elevated copper is tightly linked to sustained inflammatory phenotypes (Yang J. et al., 2025). Thus, how macrophages precisely tune copper levels to meet context-specific demands remains a central question, with emerging evidence suggesting that copper acts as a “rheostat” rather than a simple on-off switch, where subtle changes in concentration or distribution profoundly affect immune cell function.

From an evolutionary perspective, copper utilization in innate immunity is deeply conserved, reflecting an ancient arms race between hosts and pathogens (Lei et al., 2026; Zhang et al., 2024). Both prokaryotic and eukaryotic organisms have evolved sophisticated copper detoxification systems, and pathogens must counter host copper toxicity to establish successful infections. In turn, mammals have exploited copper’s toxicity as a defense mechanism, creating a dynamic battle for metal control at the host-pathogen interface (Flemming, 2023). This evolutionary history has shaped the complex copper homeostatic networks observed in modern immune cells, with macrophages playing a particularly important role. The conservation of copper’s role in immunity across diverse species underscores its fundamental importance. Notably, copper exerts dose-dependent effects on macrophage polarization: low-to-moderate copper tends to drive macrophages toward an anti-inflammatory M2 phenotype via activating STAT6 and PI3K/Akt pathways, whereas high copper concentrations trigger oxidative stress and NF-κB activation to induce pro-inflammatory M1 polarization (Huang et al., 2016). This dose-dependent regulation highlights the necessity of rigorous copper homeostasis in immune cells and suggests that the same metal can exert opposing immunological outcomes depending on its bioavailability.

Macrophages are central players in both innate and adaptive immunity, exhibiting remarkable tissue heterogeneity and functional plasticity (Hume et al., 2023). Beyond their classical role as phagocytes, macrophages regulate inflammation, coordinate tissue repair, and modulate adaptive immune responses through cytokine secretion and antigen presentation (Hamers and Pillai, 2018). Tissue-resident populations—such as Kupffer cells in the liver, alveolar macrophages in the lung, and microglia in the central nervous system—display unique transcriptional profiles and functional properties adapted to their local environment. While all macrophages share core copper homeostatic machinery, tissue-specific differences in copper handling likely contribute to these specialized functions, a hypothesis that remains largely untested. In addition to tissue heterogeneity, macrophages exhibit functional plasticity in response to environmental signals. Classically activated (M1) macrophages are pro-inflammatory, microbicidal, and glycolytic, while alternatively activated (M2) macrophages are anti-inflammatory, tissue-reparative, and oxidative. Emerging evidence indicates that copper metabolism differs substantially between these polarization states, with copper actively contributing to both the establishment and maintenance of specific activation programs (Hamers and Pillai, 2018). The M1/M2 polarization spectrum is tightly coupled to distinct metabolic signatures: M1 macrophages rely predominantly on aerobic glycolysis, while M2 macrophages favor oxidative phosphorylation and fatty acid oxidation. Copper dynamically modulates this metabolic reprogramming, forming an inseparable link among copper homeostasis, immunometabolism, and macrophage functional phenotypes.

Despite substantial progress in understanding copper biology, significant gaps remain. While the core components of copper homeostasis are well-defined in model systems, tissue-specific regulation of macrophage copper handling remains poorly understood (Leng et al., 2026). Similarly, while copper’s role in macrophage antimicrobial activity is established, its broader function in chronic inflammation and cancer immunology is only beginning to emerge, with emerging evidence revealing crosstalk among copper metabolism, cuproptosis, and TAM function (Guan et al., 2024). This review focuses on three core objectives: (1) defining the molecular mechanisms of copper homeostasis in macrophages, with particular attention to transporters (CTR1, ATP7A, ATP7B), chaperones, and signaling pathways; (2) exploring the functional interplay between copper metabolism and macrophage activation, polarization, and immunometabolism; (3) integrating mechanistic understanding with pathophysiological context to highlight translational potential. In pursuing these objectives, this review prioritizes recent work while acknowledging foundational studies. Additionally, we aim to identify critical research gaps that limit our understanding of copper-macrophage interactions, including tissue-specific regulation, single-cell dynamics of copper trafficking, therapeutic potential of copper-targeted interventions, and interplay with other trace metals. With emerging evidence linking copper dysregulation to infectious diseases, chronic inflammation, and cancer, copper homeostasis represents a promising target for therapeutic intervention (Wang et al., 2026). However, the narrow therapeutic window of copper-targeted therapies requires a deep understanding of context-specific regulation to avoid unwanted side effects. By explicitly framing these gaps, this work seeks to guide future research directions in the field and argues that copper homeostasis represents a rheostat of immune function with substantial translational potential.

## 2 Molecular mechanisms of copper homeostasis in macrophages

### 2.1 Copper transport systems: CTR1, CTR2, ATP7A

Macrophages maintain copper homeostasis through a coordinated network of transporters that control copper uptake, efflux, and intracellular distribution. The high-affinity copper transporter CTR1 (SLC31A1) is the primary mediator of copper import in most cell types, including macrophages. Structurally, CTR1 forms a homotrimeric channel that facilitates Cu(I) transport across the plasma membrane down its concentration gradient (Wang et al., 2026). In macrophages, CTR1 is constitutively expressed at the plasma membrane but rapidly internalizes in response to elevated copper levels, providing an acute regulatory mechanism to limit excessive uptake.

Notably, pro-inflammatory stimuli such as IFN-γ can upregulate CTR1 expression in M1-polarized macrophages to enhance copper import capacity. The low-affinity transporter CTR2 is mainly localized on late endosomal and lysosomal membranes, mediating copper release from vesicular compartments into the cytosol to supplement the intracellular copper pool (White et al., 2009b). In addition, CTR1 is essential for macrophage antimicrobial activity, with CTR1-deficient macrophages showing impaired copper accumulation in phagosomes and reduced killing of bacterial pathogens. This study established copper import as a critical component of the antimicrobial arsenal, setting the stage for subsequent investigations into copper’s role in immunity. While CTR1 is the primary copper importer, the low-affinity transporter CTR2 may also contribute under specific conditions, particularly in copper-rich environments. However, its specific role in macrophages remains incompletely defined.

**FIGURE 1:**
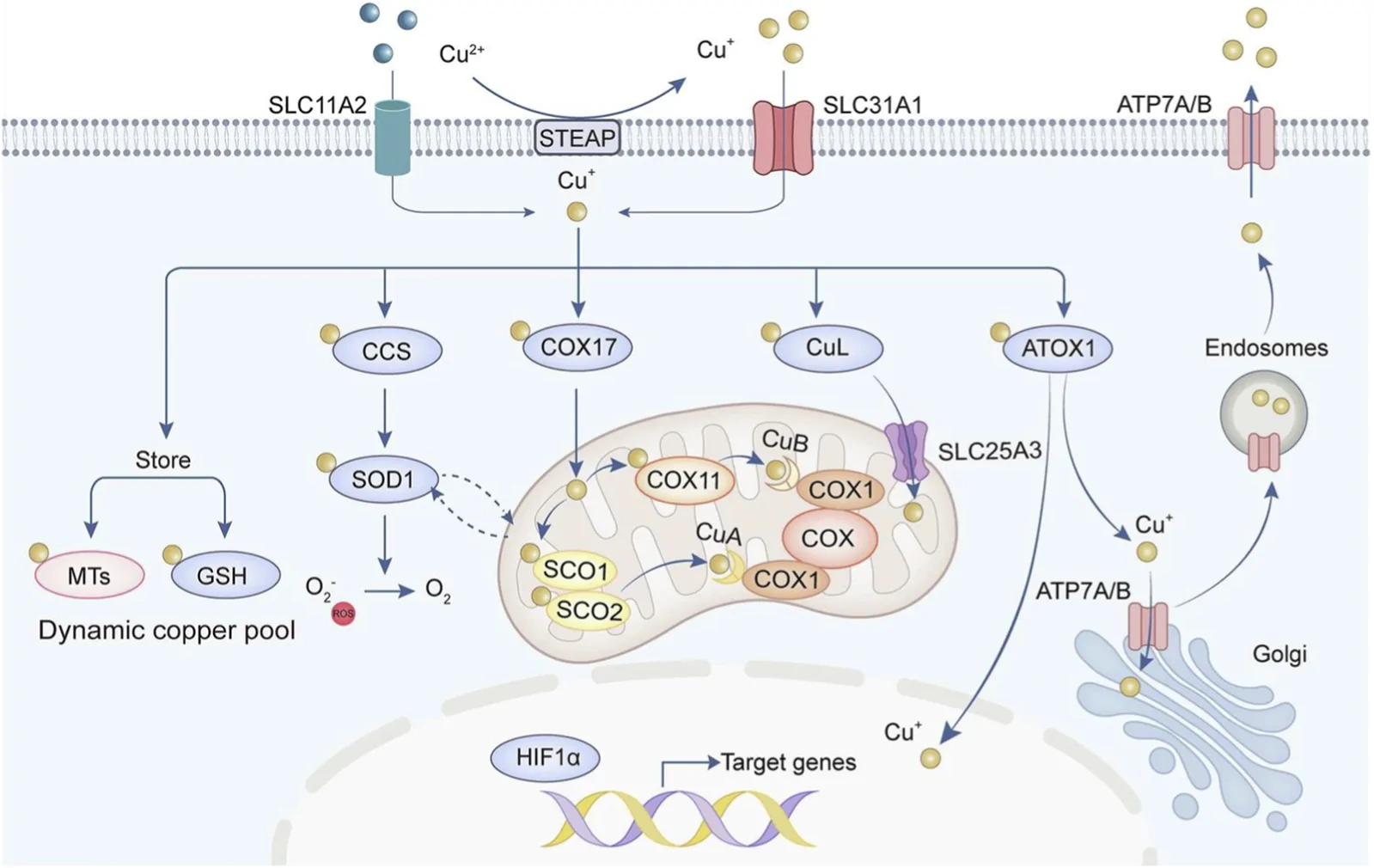
Copper transport systems and intracellular copper routing. Permission adapted from Ref (Guo et al., 2025).

On the export side, macrophages express two P-type ATPases: ATP7A and ATP7B (Kaler, 2011). Structurally homologous, these transporters use ATP hydrolysis to pump Cu(I) across membranes against its concentration gradient. In resting macrophages, ATP7A and ATP7B predominantly localize to the trans-Golgi network (TGN), where they supply copper to the secretory pathway for metalation of cuproenzymes (Figure 1; Guo et al., 2025). However, in response to elevated copper or pro-inflammatory signals, these transporters undergo dramatic relocalization. ATP7A and ATP7B exhibit distinct but overlapping tissue distributions, with ATP7A more broadly expressed and ATP7B enriched in the liver and brain. In macrophages, both transporters are present, though their relative contributions may depend on activation state. During macrophage activation, ATP7A and ATP7B relocalize from the TGN to post-Golgi vesicles and ultimately to the plasma membrane or phagosomal membrane, enabling them to export copper from the cytoplasm (Guo et al., 2025). This relocalization is regulated by multiple mechanisms, including phosphorylation and interactions with adaptor proteins. ATP7A plays a dominant role in macrophage copper trafficking and antimicrobial function: upon phagocytosis of pathogens, ATP7A translocates to the phagosomal membrane and pumps copper into the phagosomal lumen to create a toxic microenvironment for invading microbes. In contrast, ATP7B exerts a weaker effect on copper efflux in macrophages compared with ATP7A. Mutations in ATP7A and ATP7B cause Menkes disease (copper deficiency) and Wilson disease (copper overload) respectively, confirming their non-redundant physiological functions.

The subcellular trafficking of copper transporters represents a key regulatory node. In resting macrophages, the steady-state localization of ATP7A and ATP7B at the TGN is maintained by retrieval signals that return internalized transporters to the Golgi (White et al., 2009a). Upon activation, these signals are masked or modified, allowing the transporters to accumulate at their destination membranes. The precise signals controlling this relocalization remain an active area of investigation, with phosphorylation, copper binding, and possibly redox signaling all implicated. Post-translational regulation further fine-tunes transporter activity and localization. Both ATP7A and ATP7B undergo copper-dependent phosphorylation of their N-terminal metal-binding domains, which modulates their ATPase activity and trafficking. Additionally, interactions with accessory proteins can influence transporter stability and localization. For example, the copper chaperone ATOX1 interacts directly with ATP7A and ATP7B, facilitating copper transfer to the transporters and possibly regulating their trafficking (Wang et al., 2026). These post-translational mechanisms enable macrophages to rapidly adjust copper transport in response to changing conditions.

### 2.2 Copper chaperones and intracellular copper routing

Once inside the cell, copper does not exist in free form due to its potential toxicity. Instead, it is immediately bound by a network of copper chaperones that deliver it to specific destinations. Three main chaperone systems have been characterized: CCS, ATOX1, and COX17, each responsible for delivering copper to distinct cellular compartments (Palumaa, 2013). This targeted delivery ensures that copper reaches its correct destinations while minimizing the risk of off-target reactions.

**FIGURE 2:**
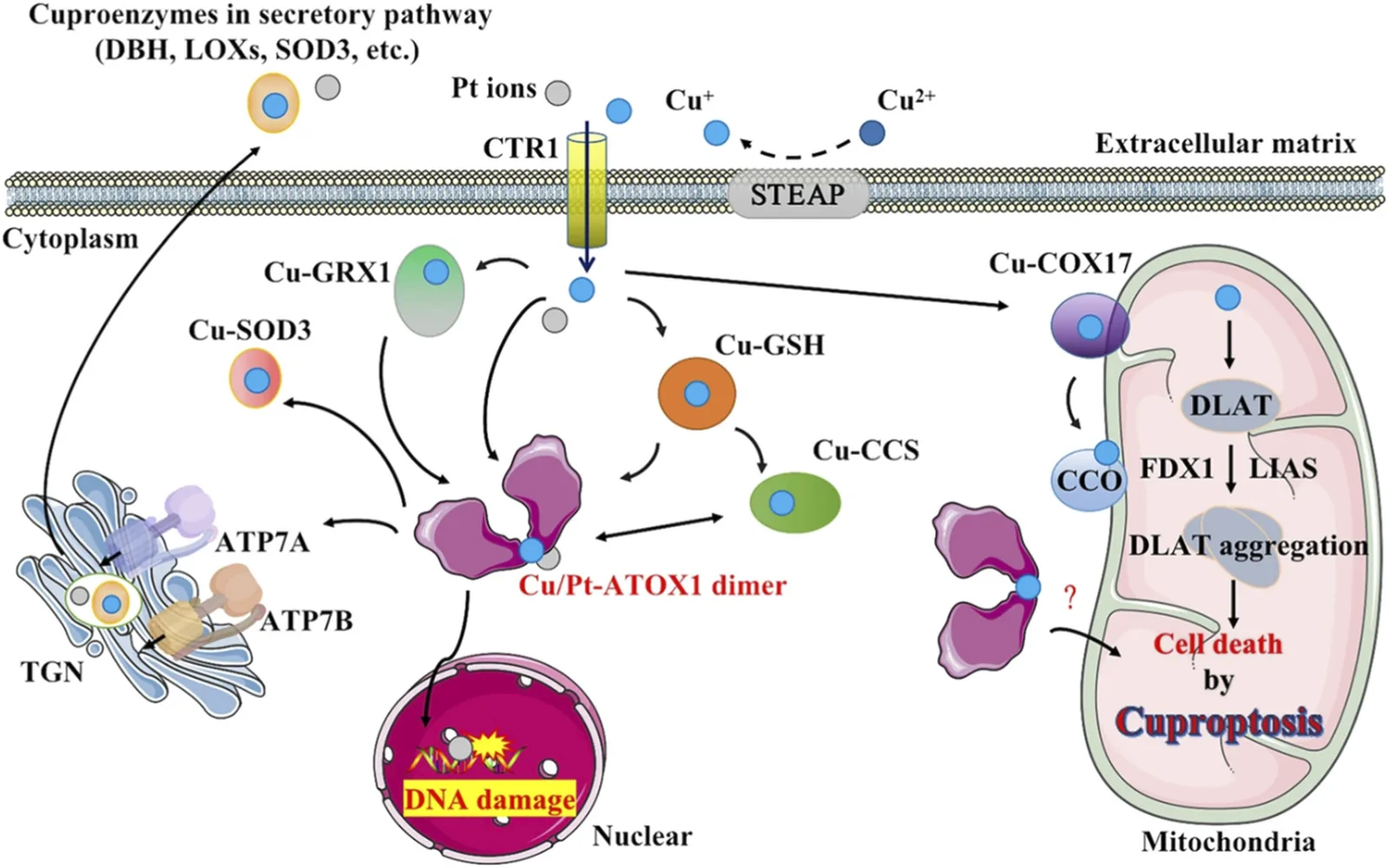
Intracellular copper trafficking pathways and functional outcomes in mammalian cells. Permission adapted from Ref (Yang et al., 2023).

CCS delivers copper to SOD1, a critical antioxidant enzyme that converts superoxide to hydrogen peroxide in the cytoplasm and mitochondrial intermembrane space (Suzuki et al., 2013). CCS binds Cu(I) with high affinity and directly interacts with SOD1, facilitating both copper transfer and the formation of SOD1’s essential disulfide bond. In macrophages, SOD1 activity is critical for managing ROS generated during respiratory burst and inflammatory signaling, making CCS function essential for macrophage homeostasis. ATOX1 delivers copper to ATP7A and ATP7B in the secretory pathway (Figure 2; Lewis et al., 2026; Yang et al., 2023). Structurally, ATOX1 contains a conserved copper-binding motif that interacts with the metal-binding domains of ATP7A and ATP7B, facilitating direct copper transfer. Beyond its role as a chaperone, ATOX1 may also function as a copper-dependent transcription factor, though this remains controversial and its relevance to macrophage biology is unclear. Regardless, ATOX1 is essential for delivering copper to the secretory pathway, a function critical for both cuproenzyme metalation and antimicrobial copper export. ATOX1 also acts as a redox sensor in macrophages, participating in the regulation of cell proliferation and DNA damage repair independent of its copper transport function. *In vitro* experiments have verified that copper ions can be exchanged between ATOX1 and CCS, revealing crosstalk among different intracellular copper routing pathways. COX17 delivers copper to the mitochondrial intermembrane space, where it is ultimately incorporated into cytochrome c oxidase (complex IV of the electron transport chain) and possibly other mitochondrial cuproproteins (Chojnacka et al., 2015). Additional mitochondrial chaperones, including SCO1, SCO2, and COX11 work downstream of COX17 to facilitate copper insertion into specific sites in complex IV. Given the central role of mitochondria in immunometabolism, copper delivery to this organelle is likely critical for macrophage metabolic reprogramming during activation.

In addition to these well-characterized chaperones, other proteins may contribute to intracellular copper routing. Metallothioneins, small cysteine-rich proteins with high copper-binding capacity, act as copper buffers that sequester excess copper (Chowdhury et al., 2019). While not strictly chaperones, metallothioneins play a critical role in copper homeostasis by maintaining a labile copper pool and protecting against copper toxicity. In macrophages, metallothionein expression is induced by pro-inflammatory signals and elevated copper, providing an additional layer of regulation. The specificity of copper chaperones ensures that copper is delivered to the correct destinations, but the mechanisms coordinating these pathways remain incompletely understood. How do chaperones “know” where to deliver copper under different conditions? Emerging evidence suggests that post-translational modifications and protein-protein interactions may regulate chaperone activity and targeting, but this remains an active area of investigation. In macrophages, activation-specific changes in chaperone function could link copper metabolism to functional outcomes.

### 2.3 Transcriptional and post-transcriptional regulation

**FIGURE 3:**
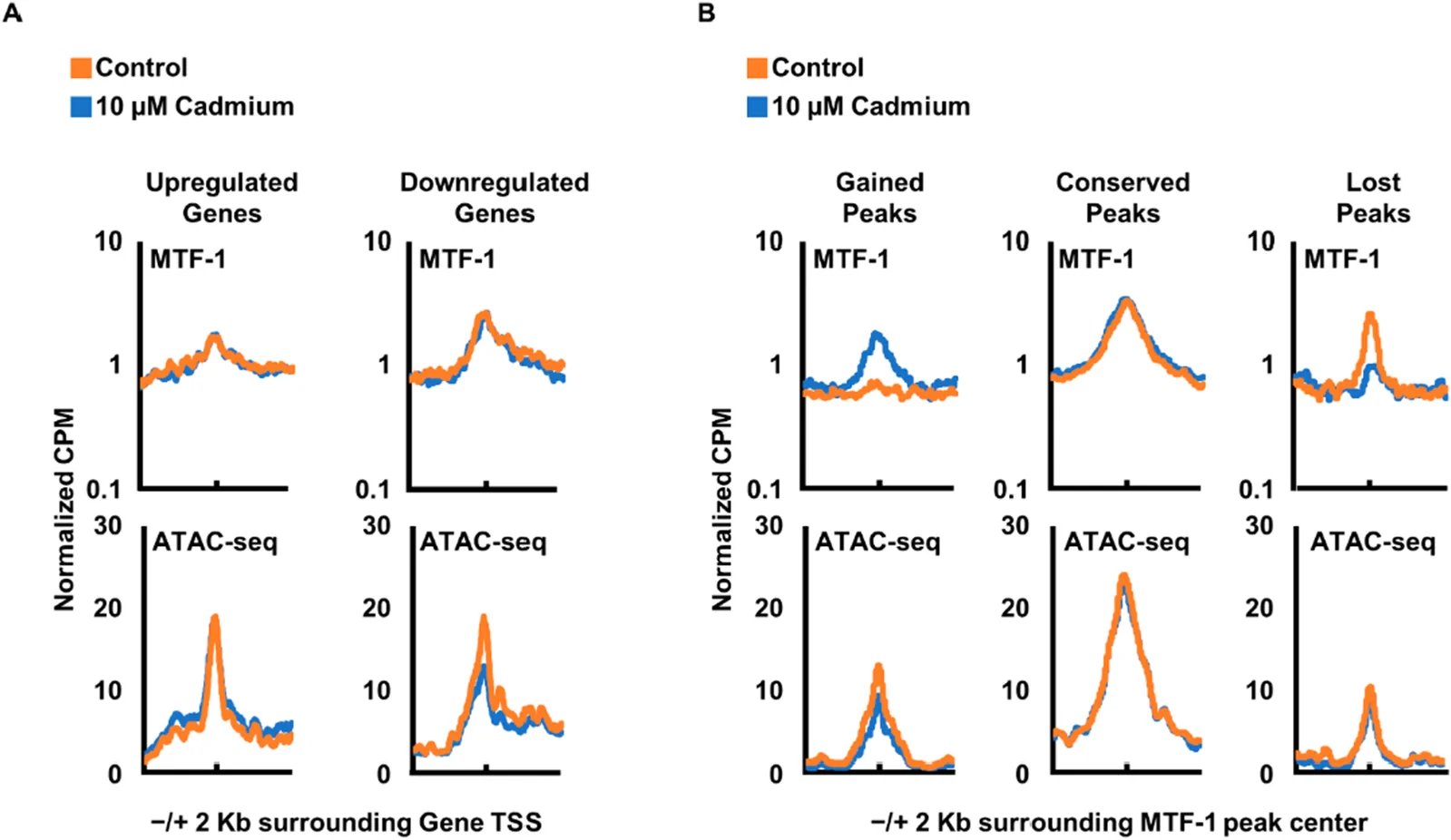
Cadmium-induced MTF-1 binding does not alter chromatin accessibility. **(A)** TSS-proximal (±2 kb) normalized CPM distributions for MTF-1 and ATAC-seq, stratified by cadmium-upregulated versus downregulated gene cohorts. **(B)** Centered meta-profiles (±2 kb) of MTF-1 occupancy and ATAC-seq accessibility across gained, conserved, and lost peak classes. (Wright and Black, 2023).

Transcriptional regulation provides a long-term mechanism for copper homeostasis. MTF-1 serves as the principal mammalian regulator, translocating to the nucleus upon copper binding to activate MRE-containing target genes, including metallothioneins. Sp1 governs adaptation to copper deficiency by upregulating uptake-related genes (Wright and Black, 2023). In macrophages, inflammatory cues also trigger MTF-1, linking immunity to copper balance. Recent genome-wide work in metal-stressed cells has refined this model by demonstrating that MTF-1 undergoes massive chromatin redistribution rather than simply increasing MRE binding (Figure 3). It vacated ∼19,000 sites while occupying ∼9,000 new ones, predominantly at distal enhancers. Released loci were enriched for FOX motifs, whereas gained regions contained AP-1 (FOS/JUN) sequences, implying a switch in transcriptional partners. Since chromatin accessibility remained unaltered, MTF-1 appears to migrate to pre-opened regulatory regions, extending its influence beyond classic metallothionein control to include genes potentially governing macrophage polarization and inflammatory responses.

In addition to MTF-1, other transcription factors contribute to copper homeostasis in a cell-type-specific manner. For example, nuclear factor kappa-light-chain-enhancer of activated B cells (NF-κB), a master regulator of inflammation, has been shown to interact with copper signaling pathways (Jing et al., 2025). Conversely, copper can modulate NF-κB activity, creating a bidirectional regulatory loop. The specific mechanisms and functional consequences of this interaction in macrophages remain an active area of investigation. Epigenetic and post-transcriptional regulation add additional layers of complexity. MicroRNAs have been shown to target mRNAs encoding copper transporters and chaperones, though few specific examples have been validated in macrophages. DNA methylation and histone modifications may also regulate copper homeostasis gene expression, though this remains largely unexplored. These regulatory mechanisms could enable stable, long-term changes in copper homeostasis in response to sustained signals, such as chronic inflammation.

To date, the identities of microRNAs that directly target core copper homeostasis genes (CTR1, ATP7A, COX17, etc.) in macrophages are still uncharacterized, representing a major unaddressed area in post-transcriptional regulation of copper metabolism. Epigenetic modifications such as DNA methylation and histone modification also participate in the long-term regulation of copper homeostasis genes during chronic inflammation.

### 2.4 Signaling pathways regulated by copper

Beyond its role as an enzyme cofactor, copper acts as a signaling molecule that modulates the activity of key signaling pathways. One of the best-characterized examples is copper-dependent regulation of NF-κB signaling. It has been demonstrated that ATP7A regulates copper-dependent NF-κB activation, with copper chelation inhibiting NF-κB activity and copper supplementation enhancing it. This effect appears to be mediated through copper’s effects on kinases and phosphatases in the NF-κB pathway, though the precise molecular mechanisms remain incompletely defined.

Copper also modulates mitogen-activated protein kinase (MAPK) signaling pathways, including extracellular signal-regulated kinase (ERK), c-Jun N-terminal kinase (JNK), and p38. As with NF-κB, copper appears to influence multiple points in these pathways, with effects depending on cell type and context. In macrophages, copper modulation of MAPK signaling likely contributes to both inflammatory activation and antimicrobial defense, though the specific mechanisms remain under investigation. Redox signaling represents another important interface between copper and macrophage function. The redox activity of copper allows it to both generate and scavenge reactive oxygen species (ROS), depending on its chemical environment and binding partners (White et al., 2009a). As a cofactor for SOD1, copper contributes to antioxidant defense, but unbound copper can generate ROS through Fenton-type reactions. This duality means that copper’s effects on redox signaling are context-dependent, with both pro-oxidant and anti-oxidant roles possible.

**FIGURE 4:**
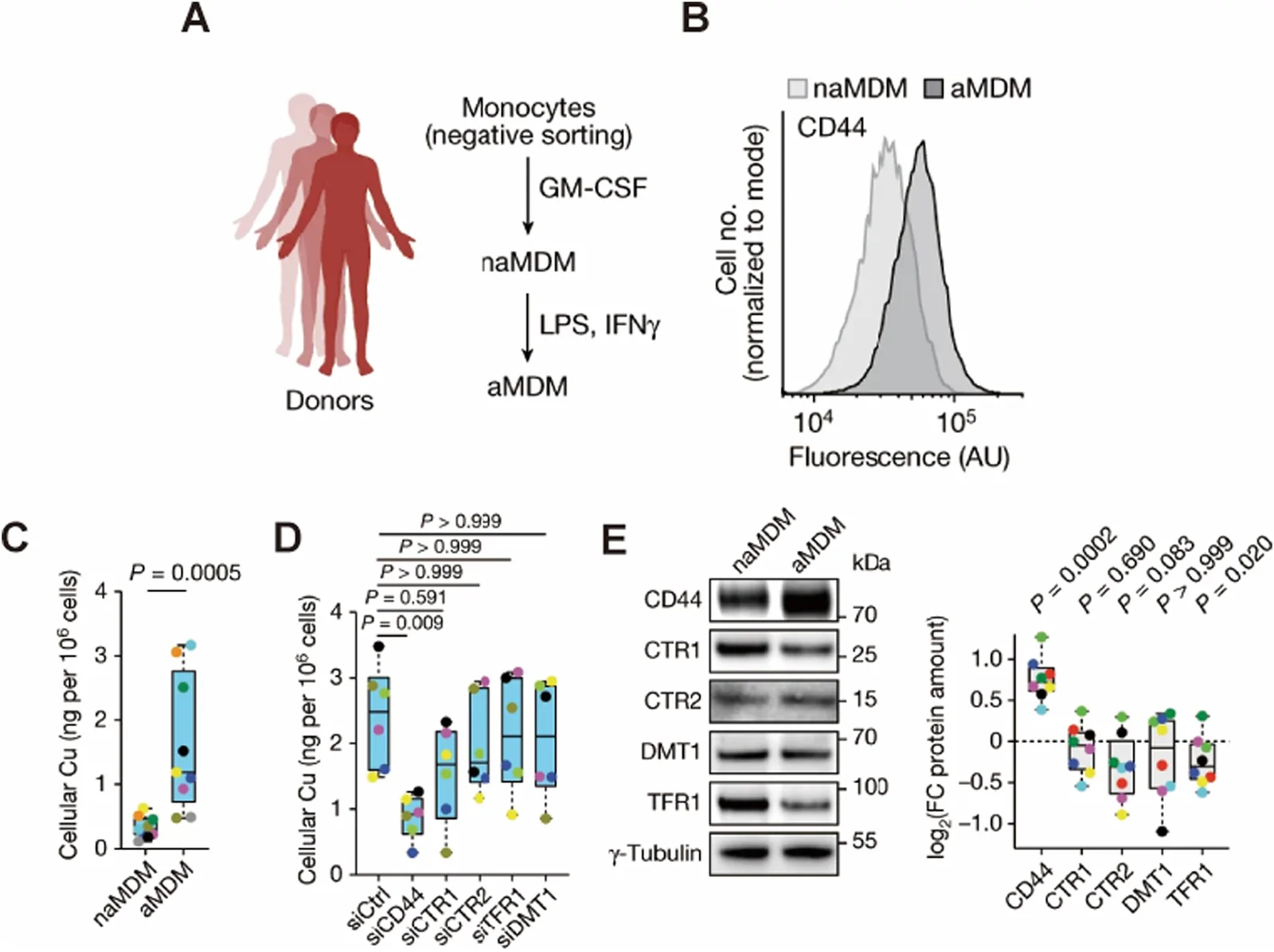
CD44 functions as a mediator of copper internalization. **(A)** Schematic of monocyte differentiation and polarization into inflammatory MDMs. **(B)** Flow cytometric assessment of CD44 surface expression on MDMs. Data represent 13 donors; AU, arbitrary units. **(C)** ICP-MS quantification of total cellular copper in MDMs (n = 9 donors). **(D)** Copper content measured by ICP-MS in inflammatory MDMs following siRNA depletion of candidate transporters and receptors (n = 6). CTR1 is encoded by SLC31A1, CTR2 is encoded by SLC31A2, transferrin receptor 1 (TFR1) is encoded by TFRC, and divalent metal transporter 1 (DMT1) is encoded by SLC11A2. siCtrl, control siRNA. **(E)** Representative immunoblots of metal transporters in MDM lysates (n = 7). FC, fold change. Permission adapted from Ref (Solier et al., 2023).

The modulation of intracellular signaling cascades by copper ions is partially mediated through the suppression of protein tyrosine phosphatase 1B (PTP1B) activity. This inhibitory action, effectively reproduced by synthetic copper coordination compounds, may represent a critical molecular pathway linking copper overload to its deleterious effects on cellular homeostasis (Wang et al., 2010). A targetable copper-signaling axis that drives inflammation has been identified. Under inflammatory stimulation, macrophages utilize CD44 to mediate cellular copper uptake via endocytosis of copper-containing hyaluronan complexes. Once internalized, copper is relayed to mitochondria via a cascade of copper chaperones including COX17, SCO1, and SCO2. The accumulated Cu(II) within mitochondria then catalyzes the oxidation of NADH, thereby sustaining NAD^+^ levels essential for driving both metabolic and epigenetic reprogramming toward a pro-inflammatory state (Figure 4; Solier et al., 2023). This pathway is pharmacologically accessible, as a dimeric metformin analogue selectively chelates mitochondrial copper and disrupts the inflammatory cascade without depleting overall copper content. Preclinical data confirm that targeting this checkpoint attenuates hyperinflammation in infection models, highlighting the therapeutic promise of interfering with copper-mediated signaling in inflammatory diseases.

## 3 Copper homeostasis in macrophage polarization and immunometabolism

### 3.1 Copper dynamics during M1 vs. M2 polarization

Macrophage activation is accompanied by substantial changes in copper homeostasis, with different activation states exhibiting distinct copper profiles. Classically activated (M1) macrophages, typically stimulated by interferon-γ (IFN-γ) and lipopolysaccharide (LPS), show increased copper uptake, elevated total copper levels, and relocalization of ATP7A/B to phagosomal membranes (White et al., 2009b) These changes support copper’s role as an antimicrobial effector, as discussed later, but also contribute to pro-inflammatory signaling. In contrast, IL-4-driven alternative activation employs a distinct copper-handling strategy centered on metallothionein 3 (MT3) (Chowdhury et al., 2019). IL-4 receptor signaling induces MT3 expression via the JAK-STAT6 cascade, and this upregulation is critical for M2 polarization. MT3 modulates labile copper distribution rather than promoting net copper accumulation, thereby facilitating the metabolic shift toward oxidative phosphorylation that underpins alternative activation. Loss of MT3 impairs M2 marker upregulation and metabolic adaptation, revealing that while M1 polarization relies on copper loading, M2 polarization depends on MT3-mediated copper sequestration to support its tissue-reparative functions.

**FIGURE 5:**
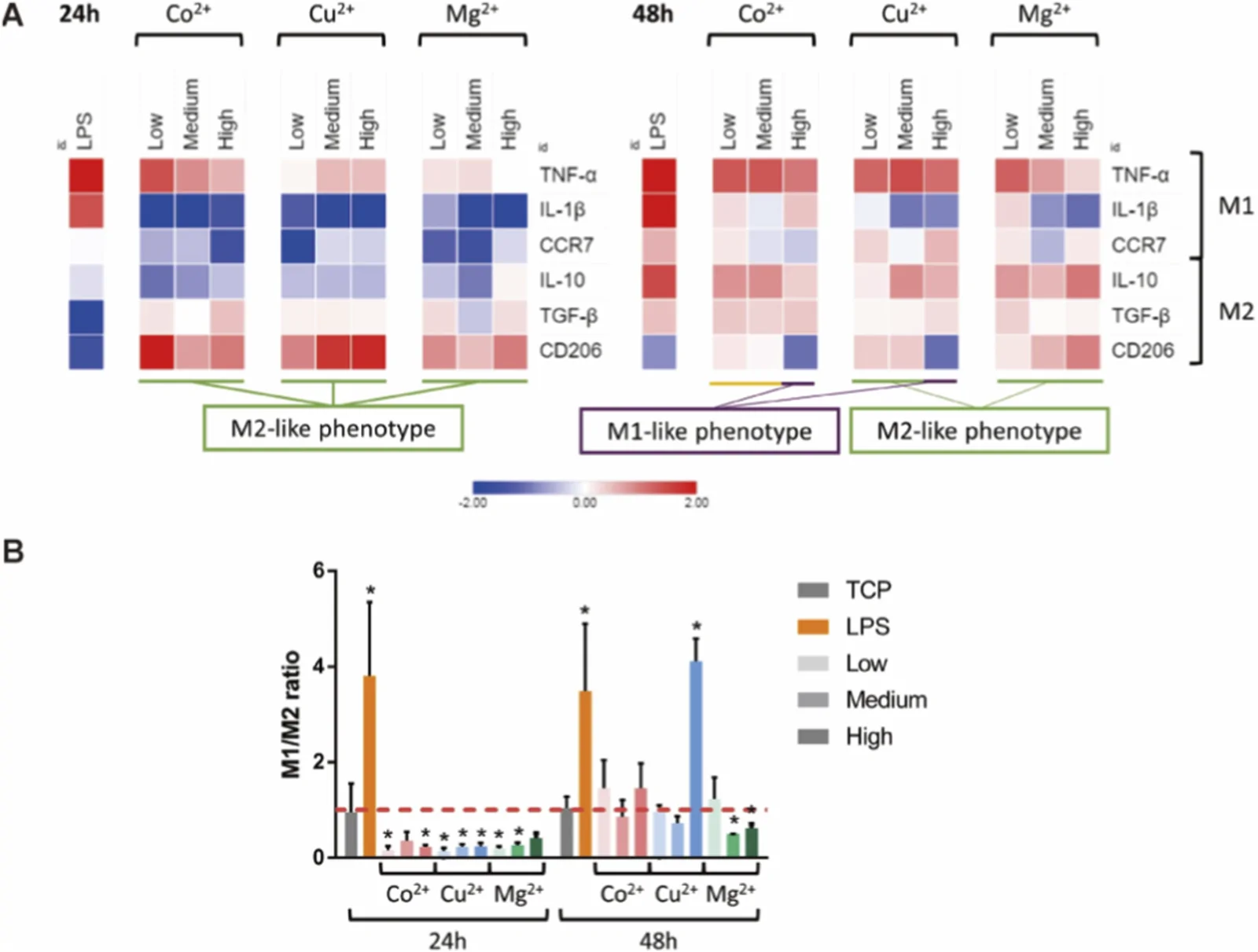
Copper accumulation and ATP7A expression in IFN-γ/LPS-stimulated macrophages. **(A)** Copper internalization assay. RAW264.7 macrophages were cultured for 24 h in serum-free medium with or without graded IFN-γ or LPS stimulation, followed by a 5-min pulse with 1 μM ^64^Cu. Uptake values were normalized to total protein and expressed as mean ± SD (n = 3). Statistical significance versus unstimulated controls was determined by Student's t-test (*, p < 0.05). **(B)** CTR1 protein abundance was evaluated by immunoblotting in whole-cell extracts from RAW264.7 macrophages stimulated for 24 h with titrated doses of IFN-γ or LPS. Band densities were normalized to tubulin and presented as fold changes relative to controls (values in parentheses). Permission adapted from Ref (Díez-Tercero et al., 2021).

A prominent feature of copper regulation in macrophages is its time- and concentration-dependent modulation of polarization. At an early stage (24 h), copper broadly suppresses pro-inflammatory markers regardless of concentration. However, by 48 h, a clear concentration-dependent bifurcation emerges: low (1 μM) elicits a mixed M1/M2 profile, moderate (10 μM) promotes a robust M2-like phenotype, while high (100 μM) drives a reversion toward the pro-inflammatory M1 state. Leire Díez-Tercero and co-workers established an *in vitro* model using PMA-primed THP-1-derived macrophages. Three Cu^2+^ concentrations—1, 10, and 100 μM—all previously validated as non-cytotoxic, were applied for either 24 or 48 h. Transcript abundances of M1-associated markers (CCR7, TNF-α, IL-1β) and M2-associated markers (CD206, IL-10, TGF-β) were quantified via qRT-PCR, and the CCR7/CD206 transcriptional ratio was employed as a composite index to gauge overall phenotypic orientation. At the 24-h time point, all tested Cu^2+^ levels suppressed M1-related transcripts while upregulating the M2 marker CD206, with CCR7/CD206 ratios falling substantially below unity—indicative of an early, uniform shift toward an M2-skewed state. By 48 h, however, concentration-dependent bifurcation emerged: 1 μM Cu^2+^ maintained CD206 expression yet concomitantly elevated TNF-α, yielding a mixed transcriptional profile; 10 μM Cu^2+^ consistently dampened IL-1β while enhancing both CD206 and IL-10, maintaining the ratio below 1.0 and thus representing the most robust condition for promoting M2 polarization; conversely, 100 μM Cu^2+^ upregulated CCR7 and sustained high TNF-α levels, driving the ratio above unity and signifying a phenotypic reversion toward the pro-inflammatory M1 state (Figure 5; Díez-Tercero et al., 2021). M1 macrophages undergo a metabolic shift from oxidative phosphorylation to glycolysis—a phenomenon known as the Warburg effect in immune cells—while M2 macrophages rely more heavily on oxidative phosphorylation (Hamers and Pillai, 2018). This metabolic switch is not simply a byproduct of activation but actively drives functional changes. Copper metabolism intersects with these pathways at multiple points, with cuproenzymes in both glycolysis and oxidative phosphorylation.

The interplay between copper homeostasis and macrophage phenotypic transitions has emerged as a critical determinant in the resolution of infected wounds. Distinct copper flux patterns have been observed during the polarization of macrophages toward pro-inflammatory (M1) or pro-resolving (M2) states, with each subset exhibiting characteristic alterations in intracellular copper content and distribution (Yang J. et al., 2025). Specifically, M1 polarization is accompanied by increased copper accumulation, which appears to sustain the oxidative burst necessary for pathogen clearance, whereas M2 polarization correlates with reduced copper levels, favoring tissue remodeling and angiogenesis. A comprehensive review by contemporary investigators systematically consolidates the current understanding of this bidirectional communication, emphasizing how copper ions modulate macrophage effector functions while simultaneously being influenced by the metabolic reprogramming that accompanies each activation state. Furthermore, the authors delineate how dysregulation of this copper-macrophage crosstalk impairs wound healing, while also discussing the translational potential of copper-based therapeutics that exploit these polarization-dependent copper dynamics to restore immune homeostasis in infected wound beds.

### 3.2 Copper as a regulator of inflammatory signaling

**FIGURE 6:**
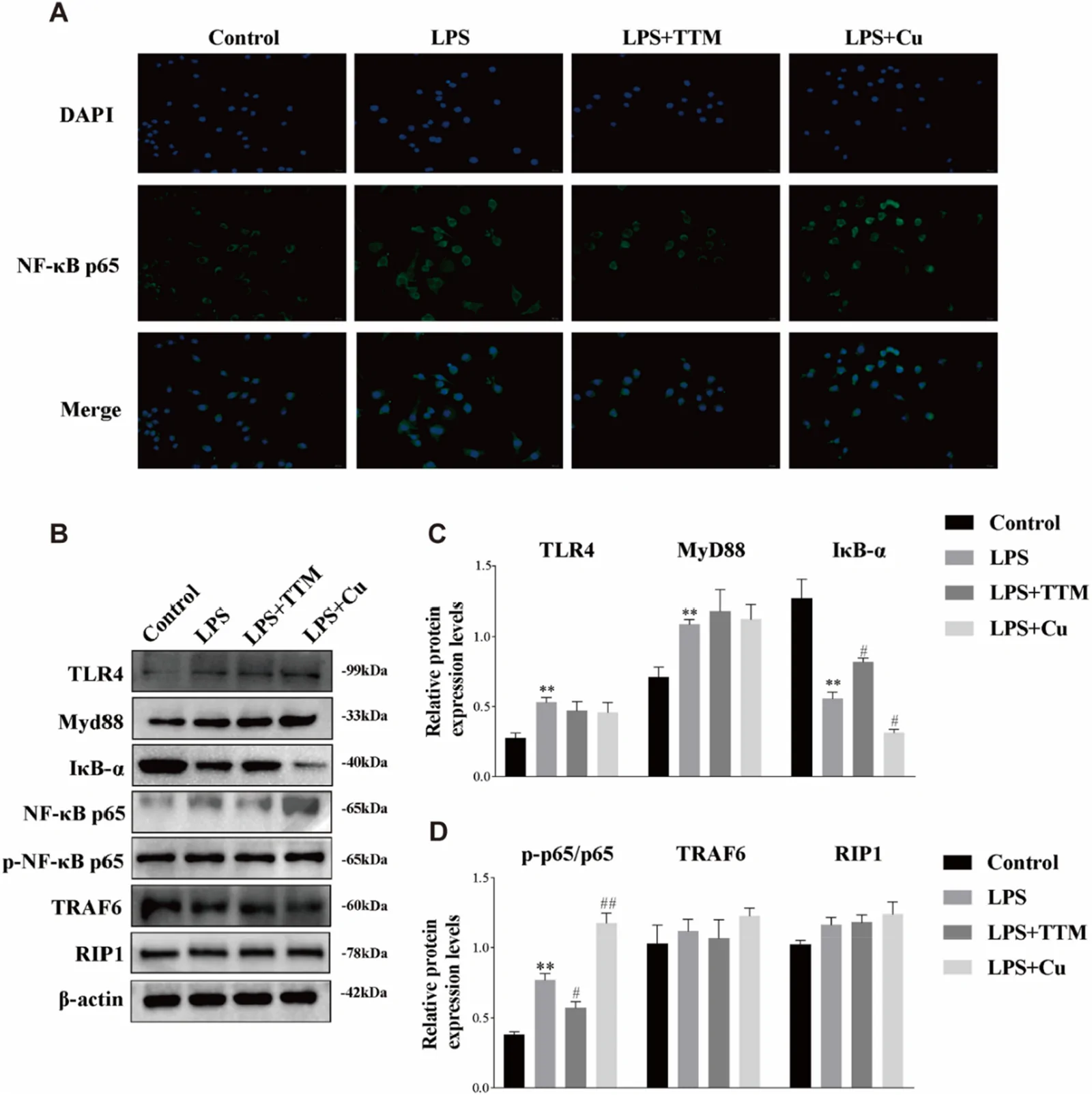
Copper promotes the activation of the NF-κB signaling axis in macrophages. **(A)** Immunofluorescence visualization of NF-κB p65 nuclear translocation in BMDMs following LPS stimulation, with or without TTM or copper co-administration (40×). **(B)** Immunoblotting detection of TLR4 (Tlr4), MyD88 (Myd88), IκB-α (Nfkbia), phosphorylated NF-κB p65, total NF-κB p65 (Rela), TRAF6 (Traf6), and RIP1 (Ripk1) in BMDM lysates under identical stimulation conditions. **(C)** The quantification of TLR4, MyD88, and IκB-α protein levels. **(D)** The quantification of p-NF-κB p65, TRAF6, and RIP1 protein levels. Permission adapted from Ref (Jing et al., 2025).

Copper regulates inflammatory signaling through multiple mechanisms, with both direct and indirect effects on key signaling pathways. As discussed earlier, copper modulates NF-κB and MAPK signaling, two master regulators of inflammation (Figure 6; Jing et al., 2025). Specifically, Matthew O. Ross et al. proposed that copper ions, at physiologically relevant concentrations, positively modulate the EGFR/MAPK/ERK signaling cascade by suppressing the activity of protein tyrosine phosphatase PTPN2. Mechanistically, Cu^+^ coordinates with the cysteine residue at the catalytic center of PTPN2, thereby attenuating its dephosphorylation function toward EGFR and consequently enhancing EGFR autophosphorylation, which in turn activates downstream MEK/ERK signaling. This cascade ultimately promotes CREB phosphorylation, driving the transcription of multiple immediate-early genes, while concurrently eliciting a moderate transcriptional repression of the copper importer CTR1, thereby establishing a negative feedback loop relevant to copper uptake regulation (Ross et al., 2024). Additionally, copper is an essential cofactor for SOD1, which modulates redox signaling by controlling superoxide levels. Since ROS are important signaling molecules in macrophages, SOD1 activity indirectly influences inflammatory signaling. Low-to-moderate copper activates STAT6 and PI3K/Akt signaling cascades, which are core drivers of M2 polarization, upregulating M2 markers such as CD206 and anti-inflammatory cytokines IL-10 and TGF-β. Excess copper triggers massive ROS accumulation, further activating the NF-κB pathway to elevate pro-inflammatory cytokines TNF-α, IL-1β, and iNOS, which are hallmarks of M1 macrophages. The copper-dependent enzyme LOX (lysyl oxidase) represents another important intersection between copper and inflammation. LOX catalyzes the crosslinking of collagen and elastin in the extracellular matrix, but it also has intracellular signaling roles (Laczko and Csiszar, 2020). While best characterized in fibroblasts, LOX is also expressed in macrophages, where it may modulate both extracellular matrix remodeling and intracellular signaling. The specific role of LOX in macrophage inflammatory signaling remains incompletely understood.

In addition to acting as a regulatory molecule of signaling pathways, the regulation of macrophage function by copper is also reflected in the effector level. Leng et al. concluded that elevated copper levels in disease-related environments can significantly enhance multiple functional outputs of macrophages, including inflammatory cytokine expression, phagocytic capacity, and bactericidal activity (Leng et al., 2026). In phagocytosis of pathogens, macrophages not only pump copper into the phagosomal cavity, but also directly kill ingested microorganisms through copper-dependent reactive oxygen species production; At the same time, copper is also involved in regulating macrophage-mediated repair processes such as tissue repair and angiogenesis. These findings further broaden the functional connotation of copper in macrophage biology-copper is not only a “varistor” for signal transduction, but also a direct participant in macrophages performing antibacterial effector functions.

Beyond its effects on individual enzymes and pathways, copper also acts as a direct signaling messenger that actively promotes the establishment of a pro-inflammatory state in macrophages (Flemming, 2023). Under inflammatory stimulation, macrophages take up copper via CD44-mediated uptake, which is then delivered to mitochondria through a sequential cascade of copper chaperones. Within these organelles, the accumulated metal catalyzes NADH oxidation, thereby driving the NAD(H) redox cycle that sustains the nucleotide pool necessary for both metabolic reprogramming and epigenetic modifications underlying pro-inflammatory gene activation. Furthermore, CD44 serves as a core key molecule for metal uptake following the activation of inflammatory macrophages. Upon activation into inflammatory macrophages, CD44 expression is significantly upregulated, accompanied by elevated intracellular accumulation of metal ions including copper, iron, manganese, and calcium. Other classical metal transporters are not involved in the metal uptake process following activation. Knockdown of CD44 antagonizes metal uptake, whereas the levels of these other metal transporters do not increase upon macrophage activation (Solier et al., 2023). This pathway, in which the metal itself serves as the pivotal mediator rather than merely a passive cofactor, illustrates a fundamentally distinct paradigm for understanding how trace elements influence immune cell fate. By directly modulating the metabolic and epigenetic landscape of macrophages, copper emerges as a critical rheostat that sets the threshold for inflammatory activation.

### 3.3 Copper and macrophage antimicrobial effector mechanisms

Macrophages employ a diverse arsenal of inducible defense mechanisms to combat intracellular pathogens, extending beyond the classical phagosome-lysosome fusion pathway. A comprehensive review by Sweet and colleagues systematically catalogues the broad spectrum of antibacterial responses that can be engaged upon macrophage activation, encompassing LC3-associated phagocytosis, metabolic reprogramming leading to antimicrobial metabolite production, lipid droplet formation, guanylate-binding protein-mediated defense, antimicrobial peptide secretion, metal ion toxicity, nutrient withdrawal, autophagy, and nitric oxide generation (Sweet et al., 2025). These pathways are triggered by pattern-recognition receptor engagement or host-derived cytokine signaling, frequently operating through inducible transcriptional programs and metabolic adaptations. Notably, copper-mediated metal toxicity is explicitly positioned within this inducible antimicrobial repertoire, operating alongside other metal-based defenses to create an intracellular environment hostile to bacterial survival. The authors further highlight that a detailed molecular understanding of these macrophage antibacterial mechanisms may inform the development of host-directed therapeutics against antibiotic-resistant infections.

Copper is a key component of the macrophage antimicrobial arsenal. Upon phagocytosis of a pathogen, macrophages rapidly mobilize copper into the phagosome, creating a copper-toxic environment that contributes to pathogen killing (Hodgkinson and Petris, 2012). This process requires CTR1-mediated copper uptake and ATP7A/B-mediated copper transport into the phagosome, creating a “copper burst” analogous to the more well-characterized respiratory burst. The mechanisms of copper-mediated pathogen killing are multiple and likely synergistic. Redox-active copper can generate ROS through Fenton-type reactions, causing oxidative damage to proteins, lipids, and DNA. Additionally, copper can disrupt iron-sulfur clusters in key metabolic enzymes, impairing bacterial metabolism. Copper can also bind to and inhibit thiol-containing proteins, disrupting their function. These multiple mechanisms make copper a potent antimicrobial agent.

**FIGURE 7:**
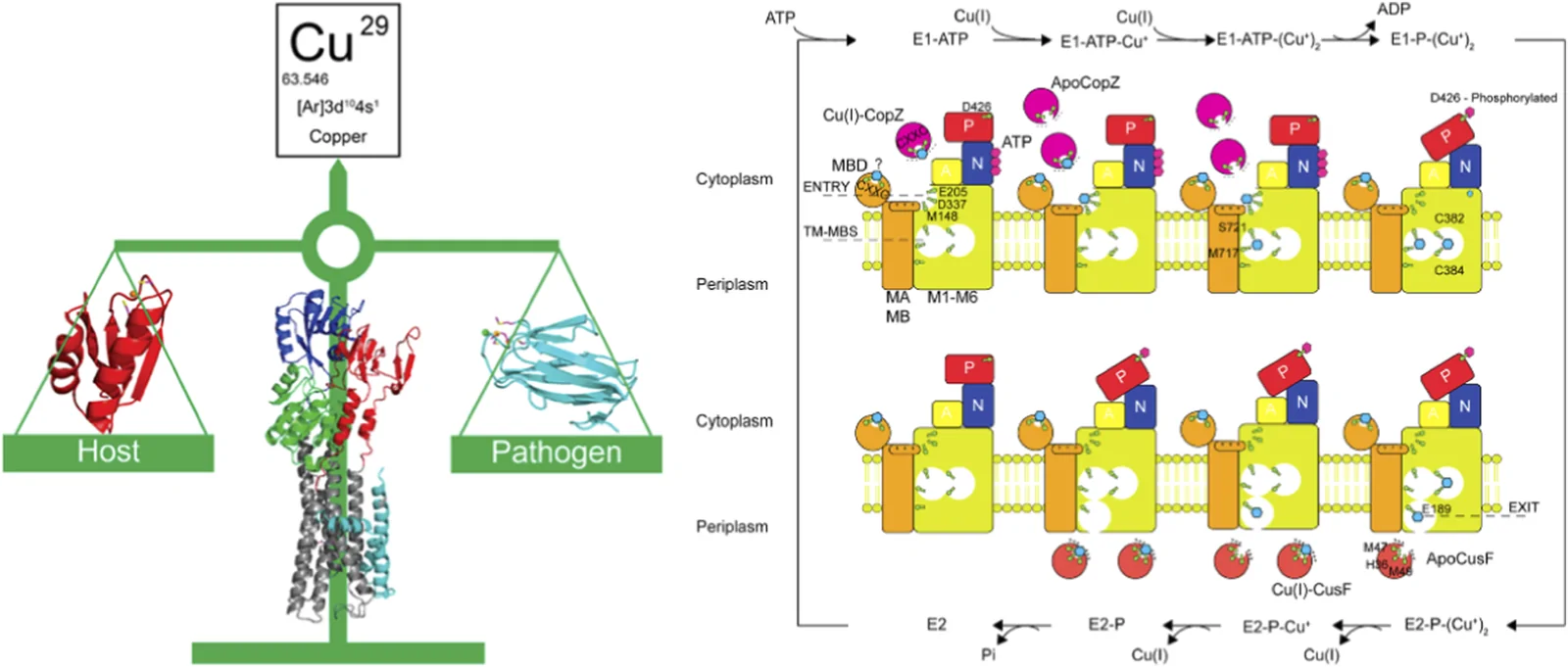
Dynamic equilibrium of copper handling systems in macrophage-bacterium interactions. Permission adapted from Ref (Fu et al., 2014).

Pathogens have evolved sophisticated countermeasures to resist copper toxicity, a testament to copper’s importance in host defense. Many bacterial pathogens express copper exporters, sequestering agents, and detoxifying enzymes that enable them to survive in copper-rich environments. Some pathogens even manipulate host copper homeostasis to create a more favorable environment, further illustrating the evolutionary arms race at the host-pathogen interface. While copper’s antimicrobial role is well-established, its relative importance compared to other antimicrobial effectors remains incompletely understood (Figure 7; Fu et al., 2014). The respiratory burst, nitric oxide production, and phagosomal acidification all contribute to pathogen killing, and their relative importance likely depends on the pathogen and context. Nevertheless, genetic evidence from both mice and humans demonstrates that copper homeostatic genes are essential for defense against at least some pathogens. Copper deficiency impairs macrophage phagocytosis, respiratory burst, and inflammatory cytokine secretion, leading to increased susceptibility to bacterial infection. The coordinated action of CTR1 and ATP7A is indispensable for phagosomal copper accumulation and subsequent pathogen clearance.

## 4 Immunological implications in health and disease

### 4.1 Copper homeostasis in infection and host defense

Copper homeostasis plays a critical role in defense against bacterial infections. *Mycobacterium tuberculosis*, the causative agent of tuberculosis, provides a well-characterized example. *Mycobacterium tuberculosis* is highly resistant to copper, expressing multiple copper detoxification systems that enable it to survive in the copper-rich environment of the macrophage phagosome (Darwin, 2015). Conversely, macrophages use copper to control *M. tuberculosis*, with copper accumulation in phagosomes contributing to bacterial killing. The balance between host copper toxicity and bacterial copper resistance likely determines the outcome of infection.

**FIGURE 8:**
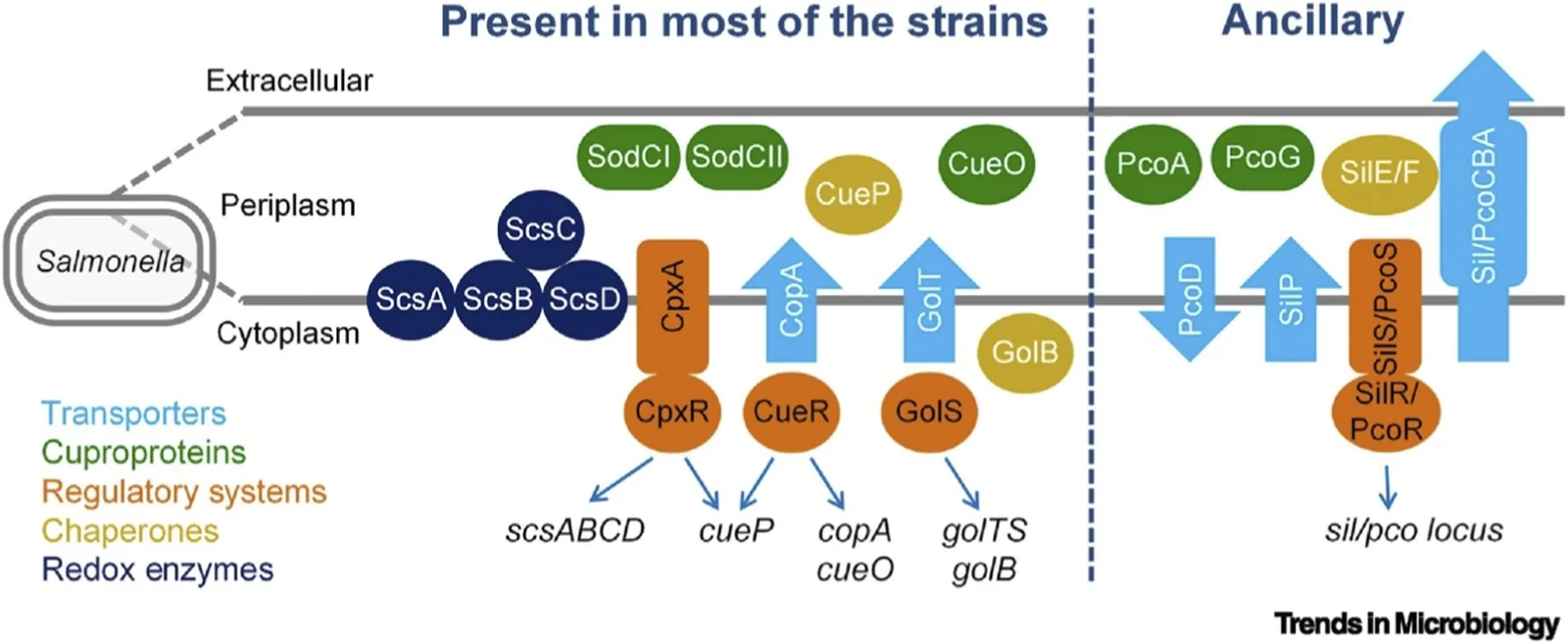
*Salmonella* Copper Homeostasis Networks. Permission adapted from Ref (Checa et al., 2021).

*Salmonella* species represent another important bacterial pathogen where copper plays a role in host defense. *Salmonella enterica* serovar Typhimurium, a common cause of food poisoning, expresses copper efflux pumps that contribute to its virulence. Mice with mutations in copper homeostatic genes show increased susceptibility to *Salmonella* infection, confirming copper’s importance in defense against this pathogen. Similar mechanisms likely operate in many other bacterial infections, though the details remain poorly characterized for most organisms (Figure 8; Checa et al., 2021). The role of copper in viral infections is less well understood, but emerging evidence suggests its importance. While viruses do not have their own copper homeostatic machinery, they can modulate host copper metabolism to create a more favorable environment for replication. Conversely, host copper homeostasis may contribute to antiviral defense through indirect mechanisms involving innate immune signaling and inflammation. The specific role of copper in viral infections remains an active area of investigation.

The interplay between copper and other metals during infection is another important consideration. Iron, zinc, and copper all play roles in host defense and are targets of pathogen manipulation (Yang Z. et al., 2025). These metals do not act independently; instead, they form a regulatory network where changes in one metal influence the homeostasis of others. In macrophages, this metal regulatory network likely contributes to both antimicrobial defense and immunomodulation, though the specific interactions remain incompletely understood.

### 4.2 Copper dysregulation in chronic inflammatory diseases

**FIGURE 9:**
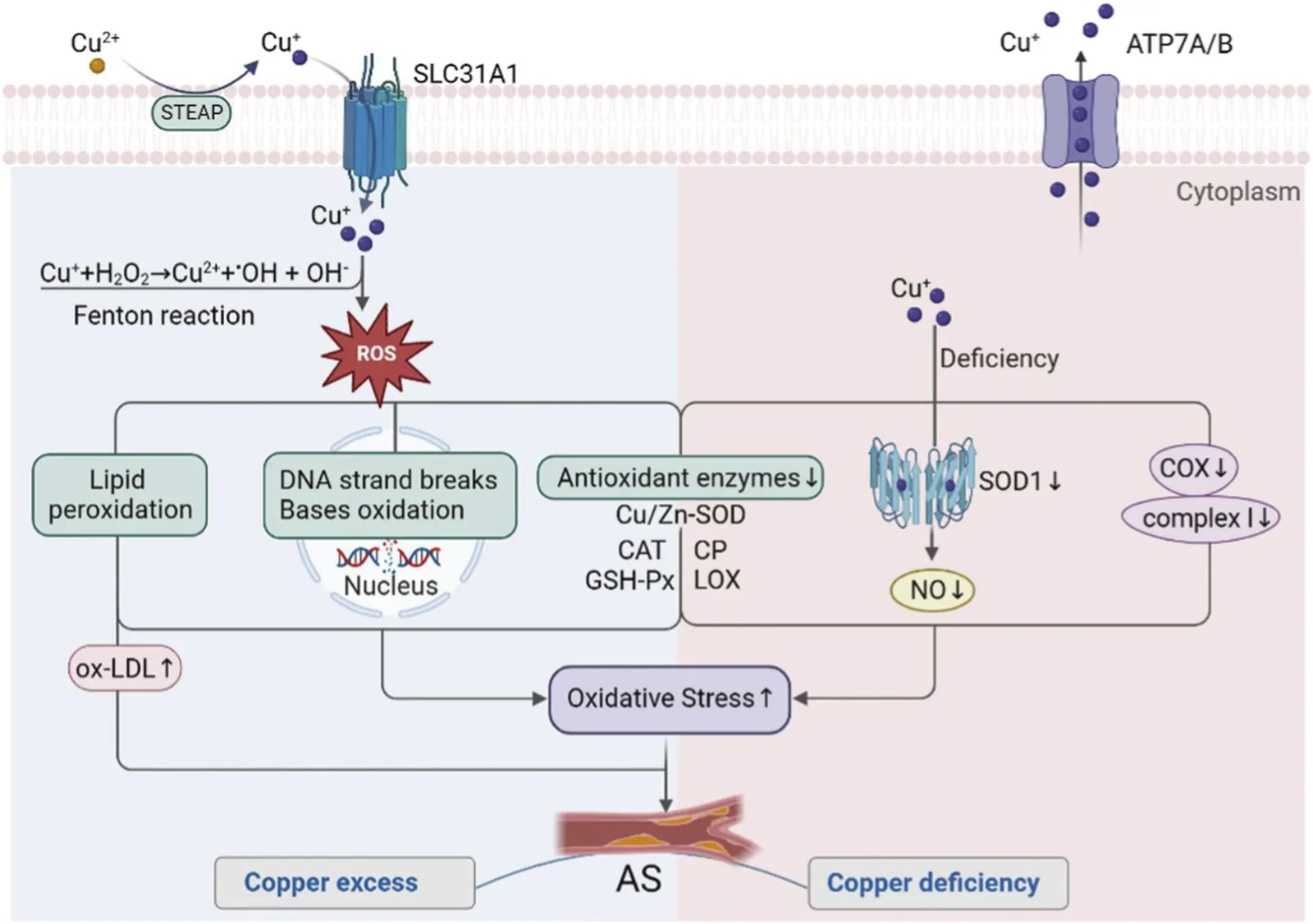
Copper dyshomeostasis promotes atherosclerosis through oxidative stress. Permission adapted from Ref (Yang et al., 2024).

Atherosclerosis and cardiovascular disease represent important contexts where copper dysregulation contributes to pathogenesis. Chronic inflammation in the arterial wall drives plaque formation, and macrophages play a central role in this process (Figure 9; Yang et al., 2024). Emerging evidence suggests that copper dysregulation in plaque macrophages contributes to disease progression, though the precise mechanisms remain debated. Copper may contribute to oxidative stress in the plaque, promoting LDL oxidation, a key early event in atherosclerosis (Qin et al., 2010). Copper exerts biphasic effects in atherosclerosis: low copper levels favor M2 macrophage polarization, which stabilizes arterial plaques, while elevated copper induces M1-dominant inflammation, exacerbating plaque instability and disease progression.

Neuroinflammation and neurodegenerative diseases represent another important context. Microglia, the resident macrophages of the central nervous system, are key mediators of neuroinflammation in diseases like Alzheimer’s and Parkinson’s (Zhu et al., 2024). Copper dysregulation has been observed in both diseases, with altered copper levels found in affected brain regions. In Alzheimer’s disease, the neuroinflammatory response mediated by microglia is intimately associated with dysregulated phenotypic polarization and disturbed copper homeostasis. Upon pathological stimulation, excess copper drives microglial transition from the homeostatic M2-like state toward the proinflammatory M1 phenotype, concomitantly triggering NF-κB signaling cascades. This activation results in the production of multiple inflammatory effectors, including nitric oxide, tumor necrosis factor, interferons, and interleukins, thereby exacerbating the neuroinflammatory milieu. Moreover, copper ions directly engage with amyloid-β (Aβ) peptides, facilitating their oligomerization and deposition into Cu–Aβ complexes. These complexes not only impair Aβ clearance mechanisms but also incite further microglial activation via oxidative injury, thus establishing a self-perpetuating vicious cycle between inflammation and Aβ accumulation. Ultimately, this concerted process accelerates cognitive deterioration and fuels the progressive neurodegenerative course characteristic of Alzheimer’s pathology (Zhu et al., 2024). Under pathological conditions in Parkinson’s disease, microglial cells drive neuroinflammation primarily through activation of the TLR4/MyD88/NF-κB signaling cascade. Upon exposure to neurotoxic agents such as MPTP or MPP^+^, microglia undergo activation characterized by elevated IBA1 expression, which in turn triggers the TLR4/MyD88/NF-κB axis. This signaling event culminates in the robust release of pro-inflammatory cytokines, including TNF-α and IL-1β, that directly inflict damage on dopaminergic neurons (Shang et al., 2025). Autoimmune diseases provide a third context where copper dysregulation may contribute to pathogenesis. Dysregulation of copper metabolism has been increasingly recognized as a contributing factor in the pathogenesis of chronic inflammatory conditions. A comprehensive review by Leng and colleagues systematically consolidates current understanding of how copper handling within macrophages influences their functional states under both physiological and pathological settings (Leng et al., 2026). The authors summarize that copper participates broadly in macrophage biology—governing antimicrobial effector functions through reactive oxygen species generation and phagosomal metal accumulation, modulating inflammatory cytokine secretion via signaling cascades, and supporting tissue reparative processes such as angiogenesis and extracellular matrix remodeling. Importantly, disruptions in copper homeostasis skew macrophage responses toward persistent inflammatory activation, a phenomenon observed in various chronic disease contexts. The review highlights that copper availability directly impacts the balance between pro-inflammatory and resolving macrophage phenotypes, with excessive copper exposure potentiating inflammatory cytokine production while copper deficiency impairs host defense and repair capacity. These insights position copper metabolic pathways as potential therapeutic nodes for mitigating inflammation-driven pathologies.

A common theme across these diverse conditions is that copper dysregulation is likely both a cause and consequence of chronic inflammation, creating a vicious cycle. Inflammation can alter copper homeostasis, while copper dysregulation can promote further inflammation. Breaking this cycle could represent a promising therapeutic strategy, though the narrow therapeutic window of copper-targeted therapies requires careful consideration of context-specific effects.

### 4.3 Copper in the tumor microenvironment

**FIGURE 10:**
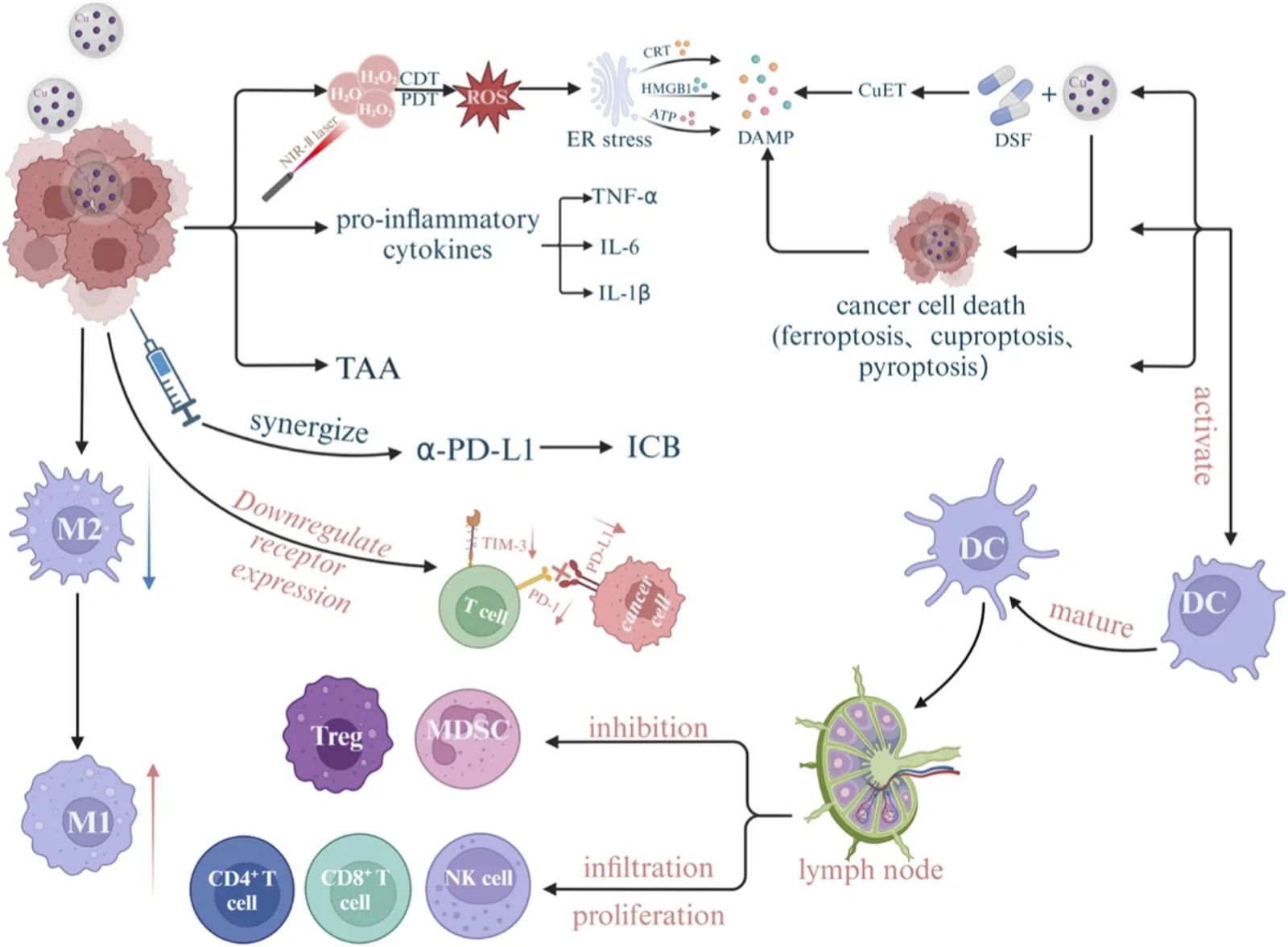
Copper nanoplatforms trigger regulated cell death and macrophage repolarization. Permission adapted from Ref (Zhang and Peng, 2025).

The tumor microenvironment (TME) exhibits a distinctive copper profile characterized by elevated metal concentrations relative to normal tissues (Tang et al., 2024). This copper-rich landscape arises from dysregulated expression of transport systems and heightened metabolic demands of proliferating malignant cells (Guo et al., 2024). A comprehensive review by Zhang and Peng systematically consolidates recent progress in copper-based biomaterials for anticancer applications (Zhang and Peng, 2025). These materials serve dual functions: as carriers for chemotherapeutics and other agents enabling precise delivery, and as direct inducers of regulated cell death, including cuproptosis, ferroptosis, apoptosis, and pyroptosis (Figure 10; Hao et al., 2026). Furthermore, a growing body of evidence has highlighted the potential of copper-based nanomedicines in modulating oxidative stress via Fenton-like reactions and multiple enzyme-mimetic activities, thereby inducing immunogenic cell death (ICD) and reprogramming the immunosuppressive TME (Xia et al., 2025). Such biomaterials can facilitate immune activation within the TME, a feature that is increasingly recognized as critical for achieving durable antitumor responses. Among immune populations influenced by copper-based interventions, TAMs represent a particularly relevant target. Copper-based nanoparticles and related formulations have been shown to promote the polarization of TAMs toward the pro-inflammatory M1 phenotype, thereby activating T cells and inducing dendritic cell maturation. This macrophage repolarization effect is corroborated by studies demonstrating that chiral copper nanomaterials and copper-based nanocarriers effectively shift macrophages from the immunosuppressive M2 state to the tumoricidal M1 phenotype, contributing to the reversal of the immunosuppressive microenvironment.

In a parallel development, an innovative nanoregulator has been engineered to simultaneously deliver copper ions and block protective autophagy, thereby amplifying cuproptotic stress while awakening antitumor immunity (Li et al., 2025). In the context of macrophage biology, copper ionophore-based platforms that induce cuproptosis and immunogenic cell death can indirectly shape macrophage function by altering the tumor metabolic landscape—for example, by reducing lactate levels that otherwise drive M2 polarization of infiltrating macrophages. This interplay between copper-mediated tumor cell death and the resultant immune remodeling underscores how strategically designed copper-based nanoplatforms can coordinate direct cytotoxicity with immunological consequences within the TME.

The therapeutic potential of copper-targeted therapies in cancer is increasingly recognized. Copper chelators like tetrathiomolybdate have shown promising results in preclinical models, inhibiting tumor growth and metastasis (Zhang F. et al., 2025; Zhang W. et al., 2025). While the precise mechanisms remain incompletely understood, modulation of TAM function likely contributes to these effects. By shifting TAMs toward an M1-like, antitumor phenotype, copper chelation could enhance antitumor immunity. However, the complexity of copper’s role in the TME means that copper-targeted therapies must be applied carefully. Copper is essential for both tumor and immune cell function, and excessive copper chelation could impair antitumor immunity (Chen et al., 2022). Understanding the context-specific roles of copper in different cell types within the TME will be critical for developing effective therapies.

## 5 Conclusion and outlooks

Copper homeostasis acts as a core regulatory module governing macrophage biology and immune function. The sophisticated network composed of copper transporters (CTR1, CTR2, ATP7A, ATP7B), intracellular chaperones (CCS, ATOX1, COX17), and multi-layered regulatory pathways enables macrophages to dynamically adjust intracellular copper concentration and subcellular distribution in response to external stimuli. Copper functions as a vital immune rheostat: its concentration exerts dose-dependent bimodal effects on macrophage M1/M2 polarization via regulating STAT6, PI3K/Akt, NF-κB and HIF-1α signaling pathways, and it is also tightly coupled with macrophage immunometabolism. In phagosomes, copper is deployed as a powerful antimicrobial weapon to eliminate invading pathogens. Meanwhile, dysregulated copper metabolism in macrophages participates in the occurrence and progression of multiple disorders, including bacterial/viral infections, atherosclerosis, neurodegeneration, autoimmune diseases, and malignant tumors. Particularly in the tumor microenvironment, copper metabolism interacts with cuproptosis to remodel the function of tumor-associated macrophages, opening up new directions for tumor immunotherapy. Looking ahead, multiple targeted research directions and technical strategies can be implemented to address existing research gaps and advance the translational application of copper-targeted interventions in macrophage-associated diseases.

First, technological innovation and multi-omics combined analysis will be the core driving force for breaking through current research limitations. Advanced imaging techniques, including X-ray fluorescence microscopy (XFM), secondary ion mass spectrometry (SIMS), and genetically encoded fluorescent copper biosensors, should be widely applied to achieve real-time, dynamic, and subcellular-resolution monitoring of labile copper pools in live macrophages. When combined with single-cell RNA sequencing, spatial transcriptomics, and proteomics, these tools can systematically characterize cell-to-cell heterogeneity in copper trafficking among different macrophage subsets, as well as the spatial distribution patterns of copper in tissue microenvironments such as atherosclerotic plaques and tumor lesions. Meanwhile, high-throughput omics platforms like RNA-seq and CLIP-seq need to be adopted to screen and validate microRNAs, lncRNAs, and RNA-binding proteins that target core copper homeostasis genes (CTR1, ATP7A, ATOX1, etc.), so as to unravel the post-transcriptional regulatory network of copper metabolism in macrophages. In addition, multi-omics approaches can also be used to explore epigenetic modifications such as DNA methylation and histone acetylation that regulate copper-related genes under chronic inflammatory conditions.

Second, genetic animal model verification and mechanism exploration are essential to clarify *in vivo* physiological and pathological functions. Macrophage-specific conditional knockout/knockin mouse lines for key copper metabolism genes (Ctr1, Atp7a, Atox1, Cox17) should be constructed. Using these models, researchers can systematically verify the roles of individual copper regulators in pathogen infection, atherosclerosis, rheumatoid arthritis, and tumor progression. Meanwhile, tissue-resident macrophage subgroups (hepatic Kupffer cells, pulmonary alveolar macrophages, central nervous system microglia) should be separately studied to reveal tissue-specific copper regulatory mechanisms and functional differences. Moreover, further research is required to explore the non-canonical functions of copper chaperones, especially the transcription factor activity and redox sensing function of ATOX1, and clarify their independent or synergistic regulatory effects on macrophage activation and polarization. The crosstalk network between copper and other trace metals (iron, zinc) in macrophage immunity also needs in-depth dissection.

Third, translational research and targeted drug development are the ultimate goals of this field. On the basis of clarified mechanisms, novel copper modulators need to be developed, including high-selectivity copper chelators, copper ionophores, and specific cuproptosis inducers. These drugs should be optimized for cell or tissue targeting to avoid systemic copper metabolic disorders caused by non-specific action. In preclinical studies, copper-targeted drugs can be combined with traditional anti-inflammatory drugs, immune checkpoint inhibitors, and other immunotherapies to evaluate synergistic efficacy, optimize medication regimens, and reduce toxic side effects. In addition, clinical retrospective and prospective studies can be conducted on patients with hereditary copper metabolic disorders (Menkes disease, Wilson disease), to analyze changes in their macrophage function and immune status, and provide human clinical evidence for copper-mediated immune regulation. Finally, cross-species comparative evolutionary research can be carried out to trace the origin and conserved mechanism of copper-dependent innate immunity, which helps to fully understand the evolutionary significance of copper-macrophage interactions.

In summary, combining cutting-edge technologies, animal model verification, standardized experiments, and translational drug research will continuously deepen the cognition of copper homeostasis in macrophages, and accelerate the transformation of basic theoretical achievements into novel diagnostic and therapeutic strategies for infectious diseases, chronic inflammation, and malignant tumors.
